# Supplementary material for: RNA origami scaffolds facilitate cryo-EM characterization of a Broccoli–Pepper aptamer FRET pair
Source: Nucleic Acids Res. 2023 Mar 31;51(9):4613–24. doi: 10.1093/nar/gkad224 (PMC10201433; doi:10.1093/nar/gkad224)
Supplement: gkad224_Supplemental_File [file gkad224_supplemental_file.pdf]

Supporting Information

RNA origami scaffolds facilitate cryo-EM characterization of a Broccoli-Pepper aptamer FRET pair

Néstor Sampedro Vallina[1,*], Ewan K.S. McRae[1,*], Bente Kring Hansen[1], Adrien Boussebayle[1], Ebbe Sloth Andersen[1,2,**]

[1]Interdisciplinary Nanoscience Center (iNANO), Gustav Wieds Vej 14, Aarhus University, DK-8000 Aarhus, Denmark.
[2]Department of Molecular Biology and Genetics, Gustav Wieds Vej 14, Aarhus University, DK-8000 Aarhus, Denmark.

*Shared first authors. **Corresponding author: esa@inano.au.dk

Supplementary Table 1: RNA designs and sequences. 2
Supplementary Table 2. Fluorescence intensities from spectrofluorometric measurements and FRET calculations. 4
Supplementary Table 3. Cryo-EM data collection, refinement and validation statistics. 5
Supplementary Fig. 1. Cryo-EM data and reconstruction of ligand bound 1,2-B12P12. 6
Supplementary Fig. 2. Cryo-EM data and reconstruction of the Apo 1,2-B12P12. 7
Supplementary Fig. 3. Goodness of fit for the aptamers modelled into the Apo and Bound reconstructions. 8
Supplementary Fig. 4. Single particle analysis workflow for the Apo 1,2-B12P12 RNA. 9
Supplementary Fig. 5. Single particle analysis workflow for the ligand bound 1,2-B12P12 RNA. 10
Supplementary Fig. 6. Full image of the SHAPE probing PAGE gel. 11

Supplementary Table 1: RNA designs and sequences.

1,2-B12P12

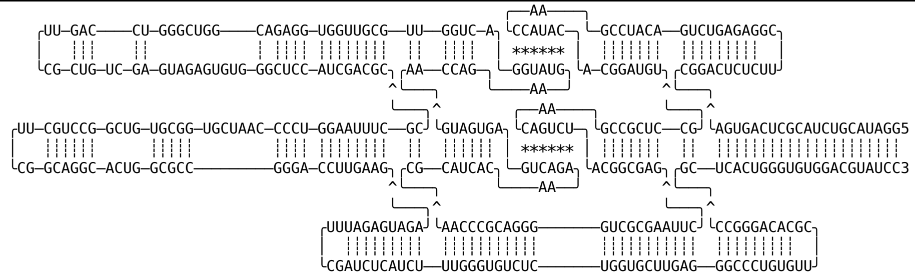

GGAUACGUCUACGCUCAGUGACGGACUCUCUUCGGAGAGUCUGACAUCCGAACCAUACACGGAUGUGCCUCGCCGAACAGUCUACGGCGAGCUUAAG
CGCUGGGGACGCCCAACGCAUCACAAAGACUGAGUGAUGAACCAGAAGUAUGGACUGGUUGCGUUGGUGGAGACGGUCGGGUCCAGUUCGCUGUCGA
GUAGAGUGUGGGCUCCAUCGACGCCGCUUUAAGGUCCCCAAUCGUGGCGUGUCGGCCUGCUUCGGCAGGCACUGGCGCCGGGACCUUGAAGAGAUGA
GAUUUCGAUCUCAUCUUUGGGUGUCUCUGGUGCUUGAGGGCCCUGUGUUCGCACAGGGCCGCUCACUGGGUGUGGACGUAUCC

1,3-B12P12

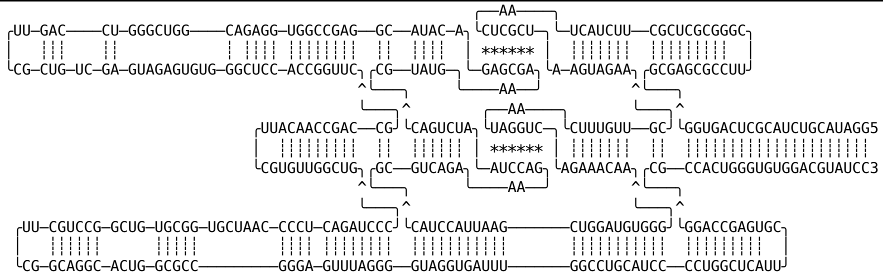

GGAUACGUCUACGCUCAGUGGGCGAGCGCCUUCGGGCGCUCGCUUCUACUAACUCGCUAAGUAGAACGUUGUUUCAAUAGGUCAGAAACAAGGGUGU
AGGUCGAAUUACCUACGCGUCAGAAAGACCUAAUCUGACCGUAUGAAAGCGAGACAUACGGAGCCGGUGGAGACGGUCGGGUCCAGUUCGCUGUCGA
GUAGAGUGUGGGCUCCACCGGUUCGCCAGCCAACAUUCGUGUUGGCUGCCCUAGACUCCCCAAUCGUGGCGUGUCGGCCUGCUUCGGCAGGCACUGG
CGCCGGGAGUUUAGGGGUAGGUGAUUUGGCCUGCAUCCCCUGGCUCAUUCGUGAGCCAGGCGCCACUGGGUGUGGACGUAUCC

1,2-B12P10

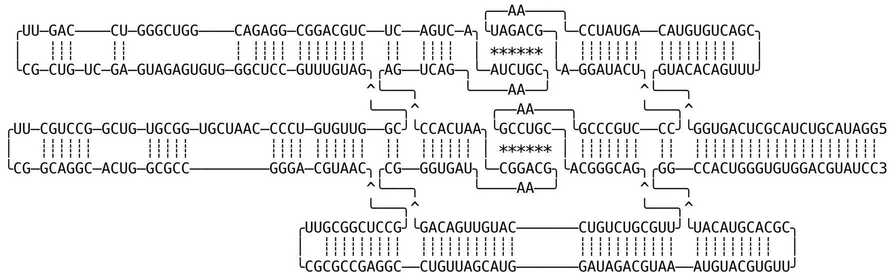

GGAUACGUCUACGCUCAGUGGGUACACAGUUUCGACUGUGUACAGUAUCCAAUAGACGAGGAUACUCCCUGCCCGAAGCCUGCACGGGCAGUUGCGU
CUGUCCAUGUUGACAGCGGGUGAUAAGCAGGCAAUCACCAGUCAGAACGUCUAACUGACUCUGCAGGCGGAGACGGUCGGGUCCAGUUCGCUGUCGA
GUAGAGUGUGGGCUCCGUUUGUAGCGGUUGUGUCCCCAAUCGUGGCGUGUCGGCCUGCUUCGGCAGGCACUGGCGCCGGGACGUAACGCCUCGGCGU
UCGCGCCGAGGCCUGUUAGCAUGGAUAGACGUAAAUGUACGUGUUCGCACGUACAUGGCCACUGGGUGUGGACGUAUCC

1,2-B12P14

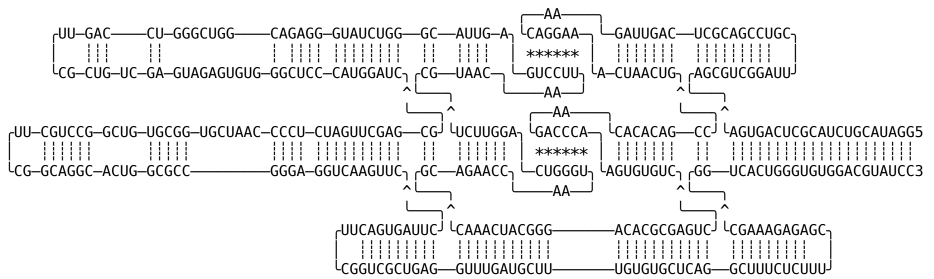

GGAUACGUCUACGCUCAGUGAAGCGUCGGAUUCGUCCGACGCUCAGUUAGAACAGGAAACUAACUGCCGACACACAAGACCCAAGUGUGUCCUGAGC
GCACAGGGCAUCAAACGCAGAACCAAUGGGUCAGGUUCUCGUAACAAUUCCUGAGUUACGGGUCUAUGGGAGACGGUCGGGUCCAGUUCGCUGUCGA
GUAGAGUGUGGGCUCCCAUGGAUCGCGAGCUUGAUCUCCCCAAUCGUGGCGUGUCGGCCUGCUUCGGCAGGCACUGGCGCCGGGAGGUCAAGUUCCU
UAGUGACUUCGGUCGCUGAGGUUUGAUGCUUUGUGUGCUCAGGCUUUCUCUUUCGAGAGAAAGCGGUCACUGGGUGUGGACGUAUCC

1,3-B12P(-34)

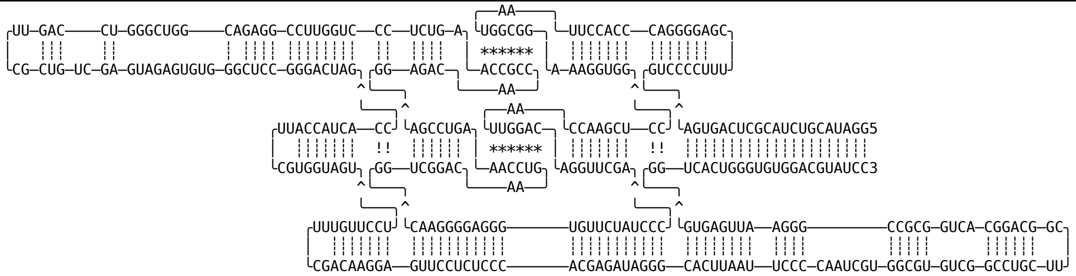

GGAUACGUCUACGCUCAGUGAGUCCCCUUUCGAGGGGACCCACCUUAAUGGCGGAAAGGUGGCCUCGAACCAAUUGGACAGGUUCGACCCUAUCUUG
UGGGAGGGGAACGGUCGGACAAGUCCAAAGUCCGAGGAGACAACCGCCAAGUCUCCCUGGUUCCGGAGACGGUCGGGUCCAGUUCGCUGUCGAGUAG
AGUGUGGGCUCCGGGACUAGCCACUACCAUUCGUGGUAGUUCCUUGUUUCGACAAGGAGUUCCUCUCCCACGAGAUAGGGCACUUAAUUCCCCAAUC
GUGGCGUGUCGGCCUGCUUCGGCAGGCACUGGCGCCGGGAAUUGAGUGGGUCACUGGGUGUGGACGUAUCC

F30-Pepper

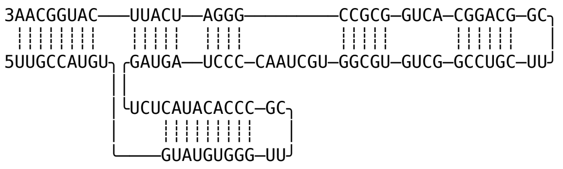

UUGCCAUGUGUAUGUGGGUUCGCCCACAUACUCUGAUGAUCCCCAAUCGUGGCGUGUCGGCCUGCUUCGGCAGGCACUGGCGCCGGGAUCAUUCAUG
GCAA

Supplementary Table 2. Fluorescence intensities from spectrofluorometric measurements and FRET calculations.

(A) Fluorescence intensities measured at excitation and emission maxima. The excitation of DFHBI-1T or HBC620 is denoted with ex_D (460 nm) or ex_A (585 nm), respectively. The emission measured of DFHBI-1T or HBC620 is denoted with em_D (505 nm), em_A (620 nm), respectively. I_D, I_A and I_{DA} refer to intensities measured in the presence of DFHBI-1T, HBC620 and both fluorophores, respectively. Data corresponds to mean value ± standard error from three pipetting replicates. (B) Calculations of donor leak (D_{Leak}), direct acceptor excitation (A_{dir}) and FRET output. Data corresponds to mean value ± standard error from three technical replicates.

A

	I_D (ex_D em_D)	I_D (ex_D em_A)	I_A (ex_D em_A)	I_A (ex_A em_A)	I_{DA} (ex_D em_D)	I_{DA} (ex_D em_A)	I_{DA} (ex_A em_A)
**1, 2-B12P12**	40230.7 ± 847	281.3 ± 4.7	1404.7 ± 22.2	28540 ± 781.2	9459 ± 225.1	7042 ± 755.9	27876.7 ± 677.3
**1, 3-B12P12**	38834.3 ± 587.4	257.3 ± 5.5	1171.6 ± 26	25582.7 ± 326.5	22492 ± 331.5	3771 ± 63.6	25386.3 ± 400.9
**1, 2-B12P10**	36290.7 ± 91	244.7 ± 15.1	1187 ± 41.2	24147 ± 679.2	8233.3 ± 110.7	5541 ± 88.4	23485.3 ± 501.2
**1, 2-B12P14**	38828.3 ± 779.9	291 ± 22.1	1310 ± 12.8	25633.3 ± 602.9	11236.7 ± 206.3	5932.3 ± 104.7	25373 ± 460.4
**1,3-B12-P(-34)**	37365 ± 1168.2	289 ± 15.1	101.7 ± 66.8	24469 ± 767.1	32691.7 ± 780.6	1332.7 ± 45.3	24602 ± 552.1

B

	D_{Leak}	A_{dir}	FRET output
**1, 2-B12P12**	0.7 ± 0.01 %	4.93 ± 0.08 %	0.372 ± 0.004
**1, 3-B12P12**	0.66 ± 0.01 %	4.58 ± 0.05 %	0.099 ± 0.0002
**1, 2-B12P10**	0.67 ± 0.04 %	4.91 ± 0.07 %	0.345 ± 0.0004
**1, 2-B12P14**	0.75 ± 0.05 %	5.12 ± 0.09 %	0.288 ± 0.001
**1,3-B12-P(-34)**	0.77 ± 0.04 %	4.08 ± 0.16 %	0.002 ± 0.002

Supplementary Table 3. Cryo-EM data collection, refinement and validation statistics.

	#1<br>Apta_FRET_Bound<br>(EMDB-14740)<br>(PDB 7ZJ4)	#2<br>Apta_FRET_Apo<br>(EMDB-17471)<br>(PDB 7ZJ5)
**Data collection and processing**		
Magnification	130000	130000
Voltage (kV)	300	300
Electron exposure (e–/Å^2)	60	60
Defocus range (μm)	-0.7 to -2	-0.7 to -2
Pixel size (Å)	0.647	0.647
Symmetry imposed	none	none
Initial particle images (no.)	729630	478981
Final particle images (no.)	150204	51278
Map resolution (Å)	4.43	4.55
FSC threshold		
Map resolution range (Å)	3.77-8.89	4.3-10.6
**Refinement**		
Initial model used (PDB code)	7PTQ - 7EOP	7PTQ - 7EOP
Model resolution (Å)	4.4 (0.143)	4.5 (1.43)
FSC threshold		
Map sharpening *B* factor (Å^2)	174	158
Model composition		
Non-hydrogen atoms	8026	7984
Nucleotide residues	374	374
Ligands	3	1
B factors (Å^2)	(mean)	(mean)
Nucleotide	234	423
Ligand	461	1012
R.m.s. deviations		
Bond lengths (Å)	0.004 (0)	0.002 (0)
Bond angles (°)	0.837 (0)	0.652 (0)
Validation		
MolProbity score	1.98	1.82
Clashscore	1.16	0.50

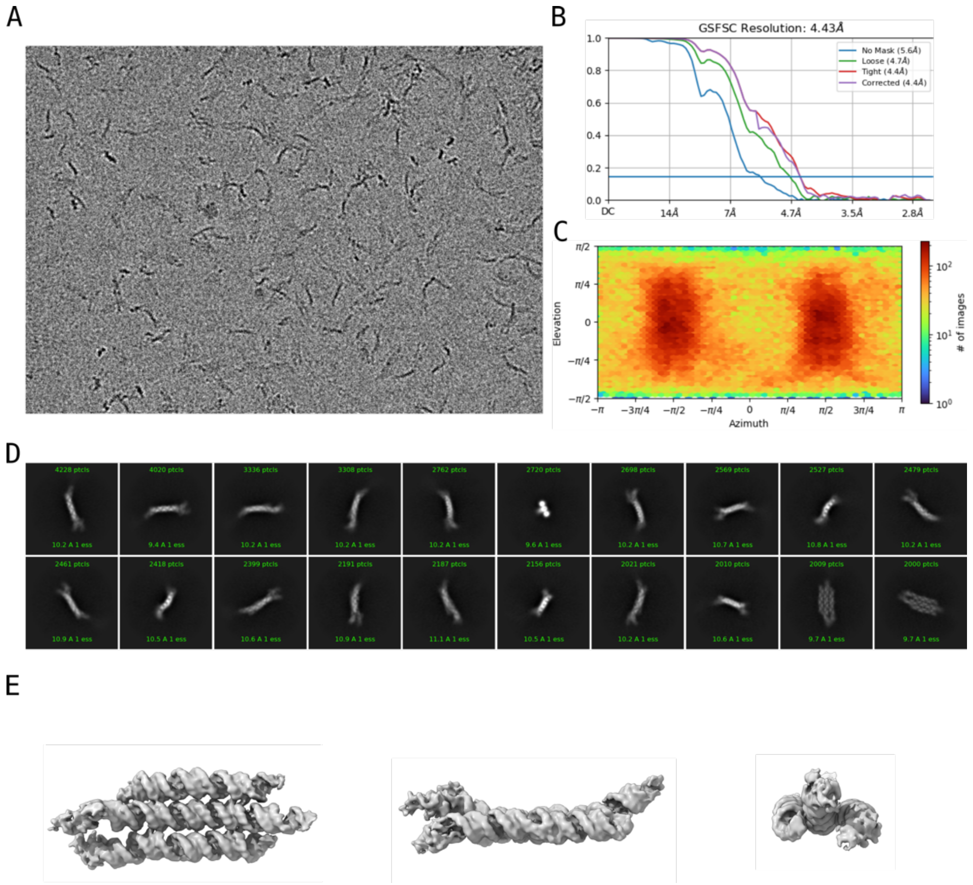

Supplementary Fig. 1. Cryo-EM data and reconstruction of ligand bound 1,2-B12P12. (A) Example cryo-EM micrograph from the ligand bound 1,2-B12P12 dataset. (B) Gold-Standard Fourier Shell Correlation for the ligand bound 1,2-B12P12 reconstruction. (C) Angular distribution of particles used in final reconstruction. (D) 2D classes from the final particle stack of the ligand bound 1,2-B12P12 dataset. (E) Three alternate views of the ligand bound 1,2-B12P12 reconstruction.

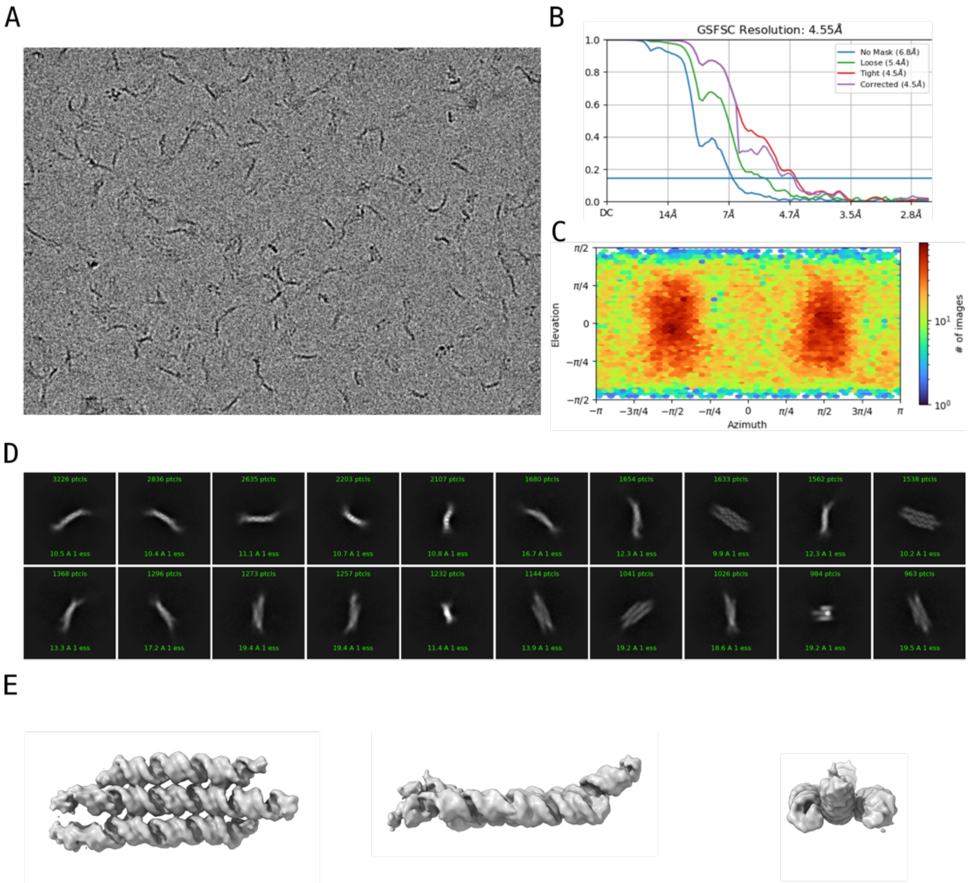

Supplementary Fig. 2. Cryo-EM data and reconstruction of the Apo 1,2-B12P12.
(A) Example cryo-EM micrograph from the Apo 1,2-B12P12 dataset. (B) Gold-Standard Fourier Shell Correlation for the Apo 1,2-B12P12 reconstruction. (C) Angular distribution of particles used in final reconstruction. (D) 2D classes from the final particle stack of the Apo 1,2-B12P12 dataset. (E) Three alternate views of the Apo 1,2-B12P12 reconstruction.

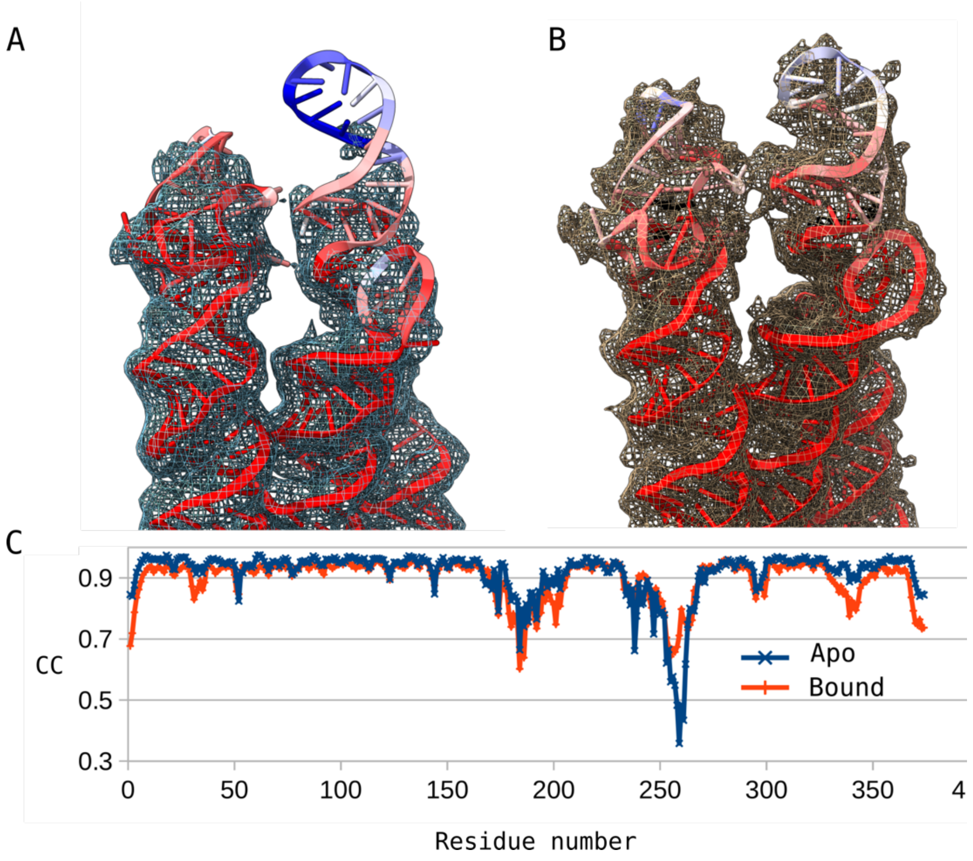

Supplementary Fig. 3. Goodness of fit for the aptamers modelled into the Apo and Bound reconstructions.

Broccoli and Pepper models colored by per-residue cross correlation coefficients (CC) for the Apo (A) and Bound (B) reconstructions. Coloring of the models is on a scale from 0.3-0.9 from blue to red, reconstructions are shown as a mesh surface with threshold level set to 0.091. (C) Per-residue CC is plotted vs residue number for the Apo and Bound models.

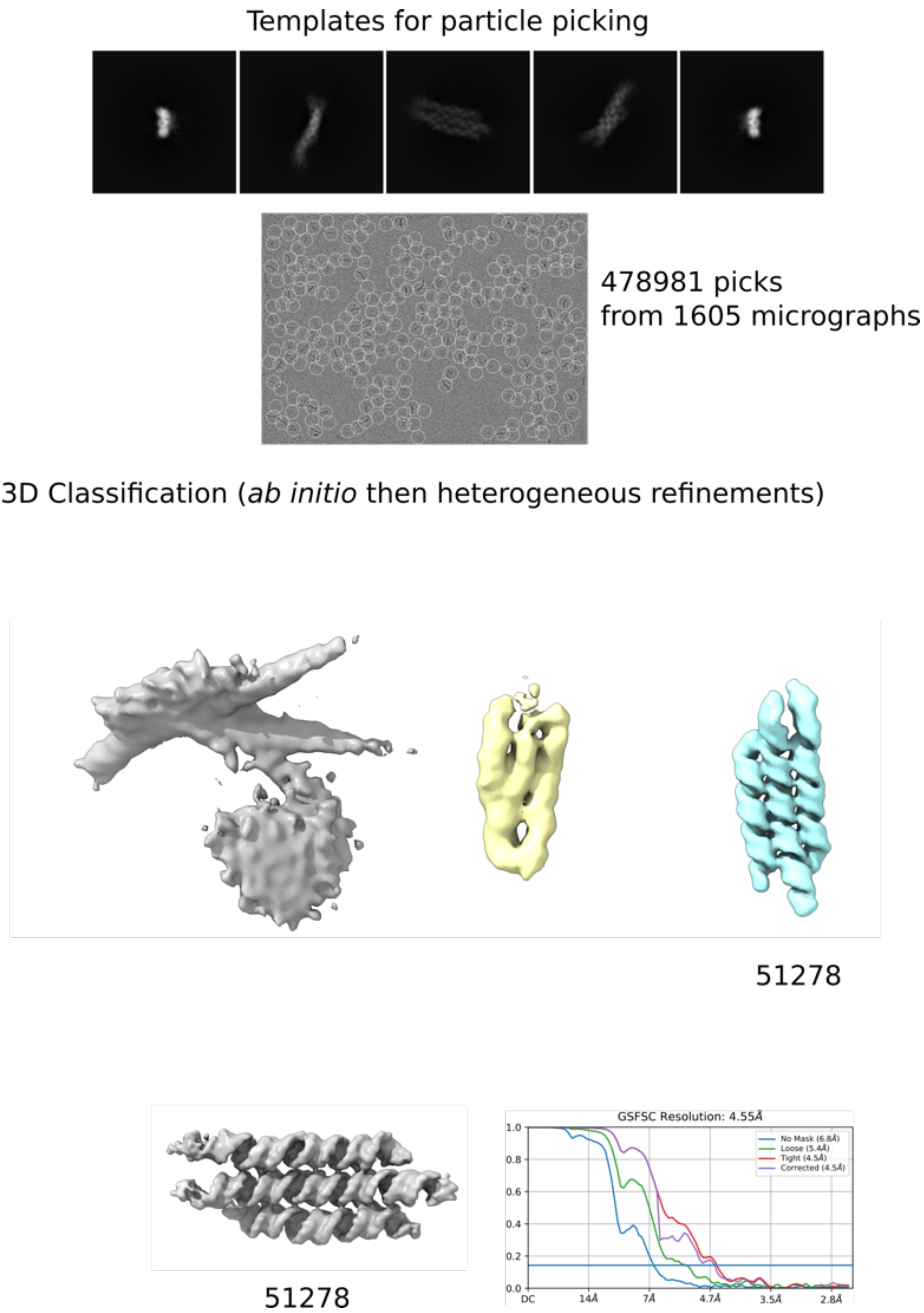

Supplementary Fig. 4. Single particle analysis workflow for the Apo 1,2-B12P12 RNA. 2D templates generated from the ab initio reconstruction obtained during a CS-Live session were used to re-pick particles from the motion and CTF corrected micrographs, followed by classification in 3D to attain the final particle stack.

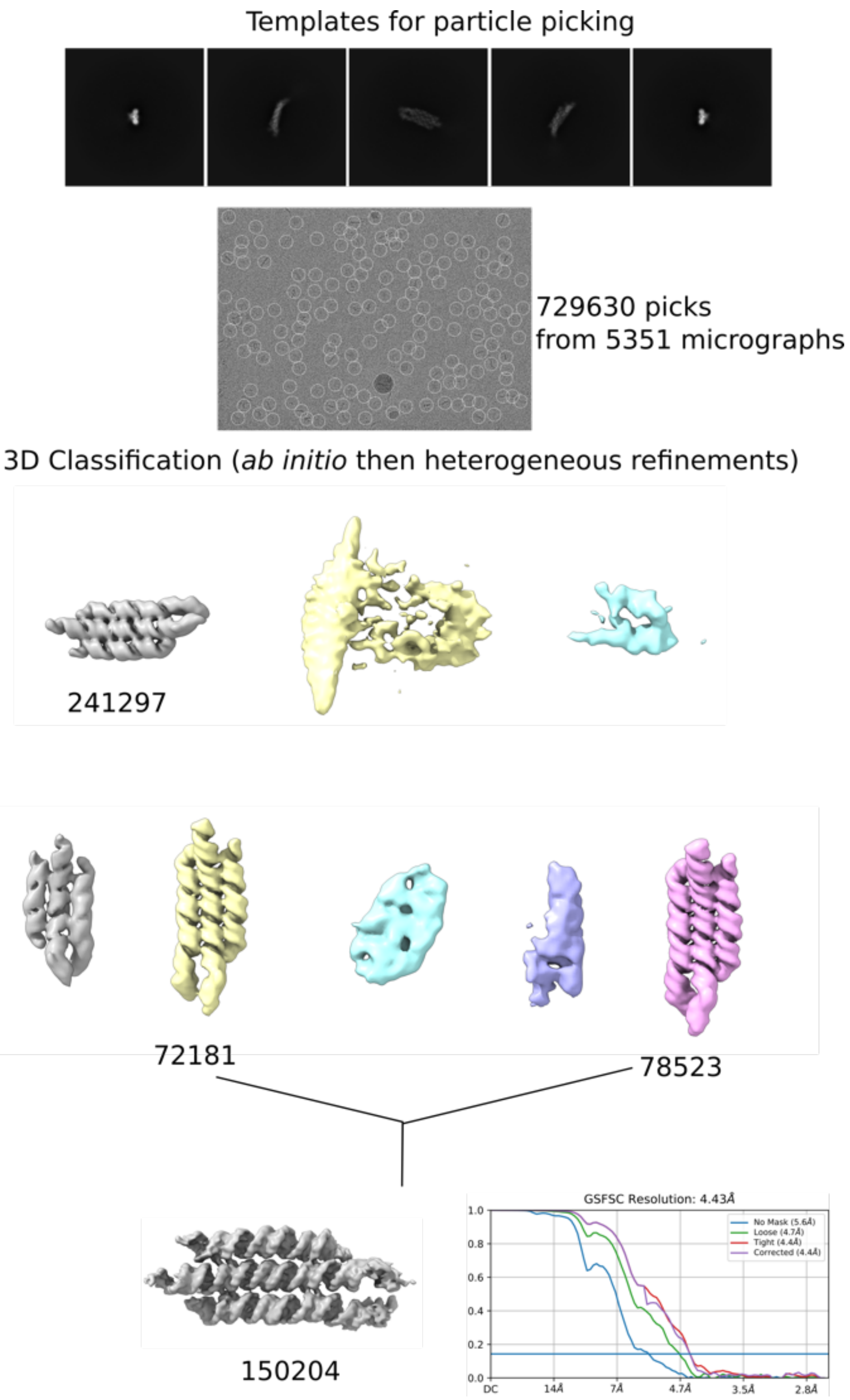

Supplementary Fig. 5. Single particle analysis workflow for the ligand bound 1,2-B12P12 RNA.

2D templates generated from the ab initio reconstruction obtained during a CS-Live session were used to re-pick particles from the motion and CTF corrected micrographs, followed by classification in 3D to attain the final particle stack.

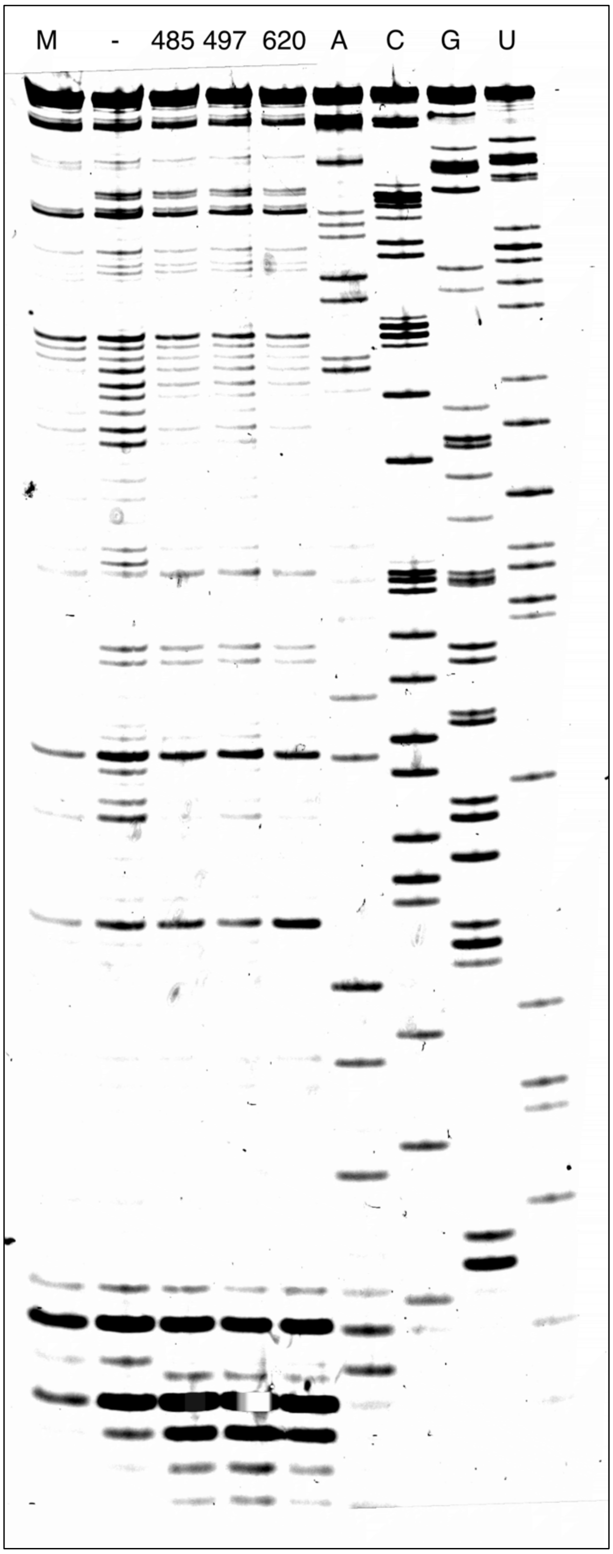

Supplementary Fig. 6. Full image of the SHAPE probing PAGE gel.
